# Acknowledgments

**DOI:** 10.1111/1759-7714.12316

**Published:** 2015-10-28

The Editors wish to thank the following reviewers of manuscripts for their contribution in 2015.

Antoine Adenis
M J Ahn
Huseyin Akdeniz
Filipe M. Andrade
Takuya Araki
Keisuke Asakura
Kazuhiro Asami
Emin Bagriacik
Alessandro Baisi
Evrim Bayman
Luca Bertolaccini
Shreekant Bharti
Andrea Billè
Johannes Bodner
R. Borie
Robert Browning
Ufuk Cagirici
L Cansever
Maria Graziella Catalano
Stephen L Chan
Jung Hyun Chang
Yeun-Chung Chang
Cheng-Yu Chen
Xiaofeng Chen
Kwan-Hwa Chi
Chun-Ru Chien
William Cho
Young-Jae Cho
Jin-Haeng Chung
Necati Citak
Maria Teresa Congedo
Marika Crohns
Joakim Crona
Yong Cui
Saurabh Dahiya
Ming-Shen Dai
Ilhan Demirci
Raj Dhupar
Takahiro Ebata
Katsura Emoto
Laura Evangelista
Serdar Evman
Elizabeth Fabre
Wentao Fang
Alper Findikcioglu
David J. Finley
Thomas Frauenfelder
Richard K. Freeman
Minoru Fukuda
Masahide Fukudo
Domenico Galetta
Jinmin Gao
Li Gao
Akiteru Goto
Ersin Günay
Shinya Hayashi
Rui Henrique
Mitsunori Higuchi
Hirotoshi Horio
Yi Hu
Chun-Chao Huang
Shiu-Feng Huang
Mohsen Ibrahim
Takaaki Ito
Yong Jiang
Zhengyu Jin
Philippe Joubert
Ozkan Kanat
Sule Karadayi
Tomoya Kawaguchi
Masaaki Kawahara
Dohun Kim
Jae Jun Kim
Tomonobu Koizumi
Parvaiz A Koul
Akihito Kubo
Yujin Kudo
Christopher Lange
Choon Taek Lee
Dae Ho Lee
Shih-Chun Lee
Hui Li
Jun Li
Serife Liman
Yajun Liu
Zhe Liu
Hao Long
Eudaldo Lopez-Tomassetti Fernandez
Vito Lorusso
Bin Lu
Effrosyni D Manali
Kamal Mansour
Teng Mao
Klazien Matter
Atul Mehta
Takahiro Nakajima
Romaine Charles Nichols
In-Jae Oh
Tatsuro Okamoto
Yusuke Okuma
Tanmay Panchabhai
Chaonan Qian
Cristian Rapicetta
Ayman Rasmy
Edward A. Ratovitski
Tommaso Ricchetti
Eduardo Rivo
Ashutosh Sachdeva
Katerina D. Samara
Haipeng Shao
Chen Shen
Kimihiro Shimizu
Tsuneo Shimokawa
Alan Sihoe
Navneet Singh
Volker Steger
Mircea Stoleriu
Kenichi Suda
Sookwhan Sung
Smrita Swamy
Federico Tacconi
Sadanori Takeo
Nagio Takigawa
Nidhi Tandon
Ali Tatlı
Naoto Tomita
Michael A. Tortorici
Ehsan Ullah
Bala basak Ustaalioglu
Alain Vergnenegre
Ko Pen Wang
Maoshui Wang
Mengzhao Wang
Shengfei Wang
Xiaoyun Wang
Yang Wang
Yi-Xiang J. Wang
Yu-Chung Wu
Yaguang Xi
Xiujie Xie
Yan Xu
Shuanying Yang
Yunfan Yang
Xue Yu
Rasa Zarnegar
Shucai Zhang
Jianying Zhou
Jiang Zhu

